# Actinomyosin Contraction, Phosphorylation of VE-Cadherin, and Actin Remodeling Enable Melanoma-Induced Endothelial Cell-Cell Junction Disassembly

**DOI:** 10.1371/journal.pone.0108092

**Published:** 2014-09-16

**Authors:** Eric Weidert, Steven E. Pohler, Esther W. Gomez, Cheng Dong

**Affiliations:** 1 Department of Biomedical Engineering, The Pennsylvania State University, University Park, Pennsylvania, United States of America; 2 Department of Chemical Engineering, The Pennsylvania State University, University Park, Pennsylvania, United States of America; University of Nebraska Medical Center, United States of America

## Abstract

During melanoma cell extravasation through the vascular endothelium, melanoma cells interact with endothelial cells through secretion of cytokines and by adhesion between proteins displayed on opposing cell surfaces. How these tumor cell associated signals together regulate the dynamics of intracellular signaling pathways within endothelial cells leading to endothelial cell-cell junction disruption is not well understood. Here, we used a combination of experimental and computational approaches to examine the individual and combined effects of activation of the vascular cell adhesion molecule (VCAM)-1, interleukin (IL)-8, and IL-1β signaling pathways on the integrity of vascular junctions. Our simulations predict a multifaceted interplay of signaling resulting from individual activation of VCAM-1, IL-8 and IL-1β pathways that is neither synergistic nor additive compared to all inputs turned on simultaneously. Furthermore, we show that the levels of phosphorylated proteins associated with actinomyosin contractility and junction disassembly peak prior to those related to actin remodeling. The results of this work provide insight into the dynamics of tumor-mediated endothelial junction disassembly and suggest that targeting proteins downstream of several interaction pathways may be the most effective therapeutic approach to reduce melanoma extravasation.

## Introduction

The spread of cancer cells from a primary tumor site to distant organs, metastasis, is one of the most devastating aspects of cancer accounting for 90% of cancer-related deaths. A key event during tumor metastasis is the extravasation of a cancer cell through the blood vessel wall [1], [2], which is mediated by both chemical and physical signals from the cellular microenvironment [3]. Following transport within the vasculature, tumor cells arrest to the endothelium and then transmigrate into the surrounding tissue, a process regulated in part by the cell-to-cell junctions of the endothelial cells. Breakdown of endothelial cell-cell junctions during extravasation is mediated by the complex interplay of cytokines secreted by the tumor cells and by adhesion between tumor cells and endothelial cells. Thus, the combined effects of both soluble and adhesive cues promote extravasation and spread of tumor cells during metastasis.

The maintenance and stability of endothelial cell-cell junctions is thought to be regulated by the balance between cell-cell adhesion and cellular contractility [4], [5]. Adhesion between neighboring endothelial cells is mediated by a variety of transmembrane cell-cell adhesion molecules including vascular endothelial (VE)-cadherin, an adherens junction protein that has been implicated in controlling vascular permeability and leukocyte extravasation [6], [7], [8], [9]. The cytoplasmic domain of VE-cadherin binds to several protein partners, including β-catenin, plakoglobin, and p120 and tyrosine phosphorylation of VE-cadherin prevents association of catenins with VE-cadherin thereby disorganizing the cadherin complex and reducing the strength of the junctions [6]. Recent studies suggest that phosphorylation of VE-cadherin is necessary but not sufficient to induce dissolution of endothelial junctions [10]; thus, the coordinated induction of multiple signaling cascades is likely key to the opening of endothelial junctions.

The cadherin-catenin complex dynamically links adherens junctions with the actin cytoskeleton and this interaction is mediated by association with α-catenin and actinin. Treatment of endothelial monolayers with hyperpermeability inducing agents leads to actin reorganization into linear, parallel bundles known as stress fibers across the cell interior [11], [12]. This actin remodeling allows for enhanced contractile forces that can contribute to the dissolution of adherens junctions. Furthermore, recent studies demonstrate that co-culture of breast cancer cells with endothelial monolayers decreases endothelial cell stiffness and increases actin cytoskeletal remodeling within endothelial cells, both of which may promote disassembly of endothelial cell-cell junctions and facilitate transmigration of tumor cells across the endothelium [13]. Cytoskeletal contractility is governed by actin and myosin which are regulated by a variety of effectors within the cell. Phosphorylation of myosin light chain (MLC) is linked to increased endothelial permeability [14], [15], [16], [17]. The phosphorylation of MLC by myosin light chain kinase (MLCK) has been studied extensively, but recently other effectors have been linked to the phosphorylation of MLC as well [4], [18]. Once MLC is phosphorylated, it activates myosin heavy chain (MHC)-II which then associates with actin to induce cellular contractility.

Melanoma cells express the ligand very late antigen (VLA)-4 (α_4_β_1_) which binds vascular cellular adhesion molecule (VCAM)-1, an integrin receptor displayed on the surface of endothelial cells [19], [20], [21]. A high expression level of VLA-4 on melanoma cells is correlated with an increase in melanoma extravasation through the endothelium [19]. We have previously shown that the VLA-4/VCAM-1 adhesion event leads to the disassembly of VE-cadherin, which facilitates melanoma extravasation [20], [22]. Activated VCAM-1 is upstream of major intracellular signaling proteins including Rac1, protein kinase C (PKC), p21-activated protein kinase (PAK), p38 mitogen-activated protein kinase (MAPK), and MLC, all of which are known to aid in endothelial cell-cell junction breakdown [18], [23], [24], [25], [26]. Furthermore, melanoma cells secrete large amounts of pro-inflammatory cytokines, including interleukin (IL)-8, IL-1β, and IL-6, and growth-related oncogene (GRO)-α which also facilitate breakdown of endothelial cell-cell junctions [26]. IL-8 acts through G-protein-coupled receptors to activate angiogenesis, proliferation and survival of endothelial and cancer cells, and migration of endothelial cells, cancer cells, and neutrophils [27], [28]. IL-8 is secreted within the tumor microenvironment and has been shown to contribute to increased endothelial permeability and to aid in the attachment of melanoma cells to the endothelium [20]. Examination of a panel of tumor cell lines revealed that invasive tumor cells express higher levels of the IL-8 receptor CXCR2 than noninvasive cancer cells and IL-8 stimulation increases tumor cell cytoskeletal remodeling and traction forces which may impact endothelial cell-cell junction breakdown during metastasis [29]. IL-1β enhances the expression of various cytokines including IL-6, IL-8, and vascular endothelial growth factor (VEGF), and adhesion molecules like intracellular adhesion molecule (ICAM)-1, therefore facilitating adhesion and extravasation events [30]. Moreover, IL-1β also induces intracellular signaling via the nuclear factor (NF)-κB and MAPK pathways, which can lead to a number of other cellular actions [31].

Melanoma cell extravasation is a complex process that can be summarized into three steps: adhesion to the endothelial cell surface, retraction of the endothelial cells, and tumor cell invasion through the endothelium into the surrounding tissue [2]. All three of these steps are regulated by an intricate network of intracellular signaling cascades within endothelial cells that communicate via crosstalk. It is becoming increasingly recognized that complex systems like these need to be analyzed holistically, and not in a reductionist manner. Thus, due to the challenge of examining the dynamics of multiple signaling pathways simultaneously in co-culture experiments alone, for the present study we integrated both experimental and computational approaches to elucidate the roles of VCAM-1, IL-8, and IL-1β in junction disassembly, actin remodeling, and actinomyosin contractility in melanoma-induced endothelial cell-cell junction disruption. Our simulations reveal that phosphorylation of MLC is achieved before peak VE-cadherin phosphorylation suggesting the induction of endothelial contractility pathways may precede reduced adhesion strength of the adherens junctions and the dissolution of cell-cell junctions. Additionally, the dynamics of MLC phosphorylation within endothelial cells is not simply additive with the simultaneous activation of VCAM-1, IL-8, and IL-1β signaling cascades, but rather these cascades seem to exert antagonistic effects during the later stages of gap formation. Interestingly, VCAM-1 is able to recapitulate the effect on VE-cadherin phosphorylation that all inputs have when activated together, suggesting the importance of VCAM-1 in regulating junction dynamics. VCAM-1 also appears to be the most important input for actin remodeling, though IL-8 and IL-1β do activate it to a lesser extent. These results indicate that junction disruption occurs in a multi-step manner with both soluble factors and binding events as actors in the process.

## Materials and Methods

### Cell culture

Human umbilical vein endothelial cells (HUVECs) were obtained from American Type Culture Collection (ATCC) and cultured in F-12K medium supplemented with 10% fetal bovine serum (FBS; Atlanta Biologicals), 100 units/ml penicillin-streptomycin (pen-strep; Biofluids, Inc.), 30 µg/ml endothelial cell growth supplement (ECGS; Sigma), and 50 µg/ml heparin (Mallinckrodt Baker, Inc.). A2058 melanoma cells were obtained from ATCC and cultured in DMEM/F-12 (Life Technologies) supplemented with 10% FBS and 100 units/ml pen-strep. All cells were grown at 37°C and 5% CO_2_ in a humidified incubator.

### Endothelial-melanoma co-culture

HUVECs were seeded at 3×10^5^ cells to 25-mm diameter glass coverslips coated with 1 µg/ml human fibronectin (BD Biosciences) for all experiments. When 95–99% confluent, monolayers were incubated with F-12K media supplemented with 2% FBS for 12 hours prior to co-culture. For co-culture, A2058 melanoma cells were lifted from tissue culture dishes using a thin coating of 0.5% trypsin (Life Technologies). The A2058 cells were then suspended in fresh medium and rocked for 1 hour in a 37°C incubator. A2058 melanoma cells were then plated at 1.8×10^5^ cells/cm^2^ to the endothelial monolayers. Endothelial cell-cell junction integrity was monitored following 45 minutes of co-culture.

### Pharmacological inhibitors

HUVEC monolayers were incubated with the following inhibitors or carrier solution for 30 minutes prior to co-culture with A2058 melanoma cells: blebbistatin (25 µM; Sigma), cytochalasin D (0.01 µg/ml; Tocris Bioscience), PP1 (0.17 µM; Sigma), ML-7 (5 µM; Sigma), or dimethyl sulfoxide (DMSO; Sigma). Following incubation with inhibitors, HUVEC monolayers were rinsed once with F-12K/2% FBS culture medium and were then co-cultured with A2058 melanoma cells.

### Immunofluorescence staining

Following co-culture, HUVEC monolayers were gently rinsed once with 1× phosphate buffered saline (PBS) and fixed with 4% paraformaldehyde for 15 minutes at room temperature. Cells were then washed with PBS, permeabilized with 0.3% Triton X-100 and blocked with 5% goat serum (Sigma). Samples were then incubated with VE-cadherin primary antibody (D87F2; Cell Signaling Technologies) overnight at 4°C. The next day samples were thoroughly rinsed with PBS to remove unbound primary antibody, incubated with AlexaFluor secondary antibodies (Life Technologies), and then counterstained with Hoechst 33342 (Life Technologies) to visualize cell nuclei. Coverslips were mounted to microscope slides using Fluoromount G (Southern Biotechnology) prior to imaging.

### Microscopy and analysis

Samples were imaged with a 40× air or 100× oil objective (N.A. = 1.3) using a Nikon Eclipse Ti-E inverted fluorescence microscope equipped with a Photometrics CoolSNAP HQ^2^ charge coupled device camera. Images were taken from 9 randomly chosen locations within the monolayer. Gaps between endothelial cells were quantitatively measured from fluorescence images of VE-cadherin using ImageJ software (NIH) as the ratio of pixels within all monolayer gaps to the total number of pixels in one image.

### Statistical analysis

Statistical analysis was performed with KaleidaGraph 4.1 software (Synergy) using analysis of variance (ANOVA) followed by a Tukey Honestly Significant Difference (HSD) post-hoc test. Differences were considered significant for p<0.05. Values are reported as mean ± standard error of the mean. Three independent replicates were performed for all experiments.

### Modeling signaling cascades important to melanoma-induced endothelial cell-cell junction dissolution

An intracellular signaling network was created to connect the VCAM-1, IL-8, and IL-1β inputs and their respective signaling cascades to factors that are responsible for regulating endothelial junction breakdown. The interactions were altered to represent enzyme kinetics and a system of ordinary differential equations was developed using these kinetics to construct a model using a MATLAB systems biology toolbox developed by Schmidt and coworkers [32]. The interactions between nodes were represented by a kinetic expression to describe its respective rate and each node was given an initial concentration (Tables S1 and S2). Modeling was performed as described in previous work published by our group with the addition of the c-Src signaling pathway [26]. The model was built using the following assumptions, which supplement the model's previous assumptions:

1. Kinetic parameters and initial concentrations were obtained from literature and experimental data. If the specific value of the concentration or parameter was not available, then they were set to 1 µM and 0.1 µM^−1^ s^−1^, respectively [33]. Simultaneous activation of all three inputs (VCAM-1, IL-8, and IL-1β) was assumed to mimic tumor cell activation of the endothelium.
2. For time course simulations, the time at which the peak protein concentration occurred was assumed to be the optimal activation of that protein.
3. Due to the complexity of inhibitory kinetics and the lack of knowledge of the type of inhibitions on the proteins in the model, inhibitory reactions were neglected.

## Results

### Melanoma cells induce breakdown of endothelial cell-cell junctions

**Figure 1:**
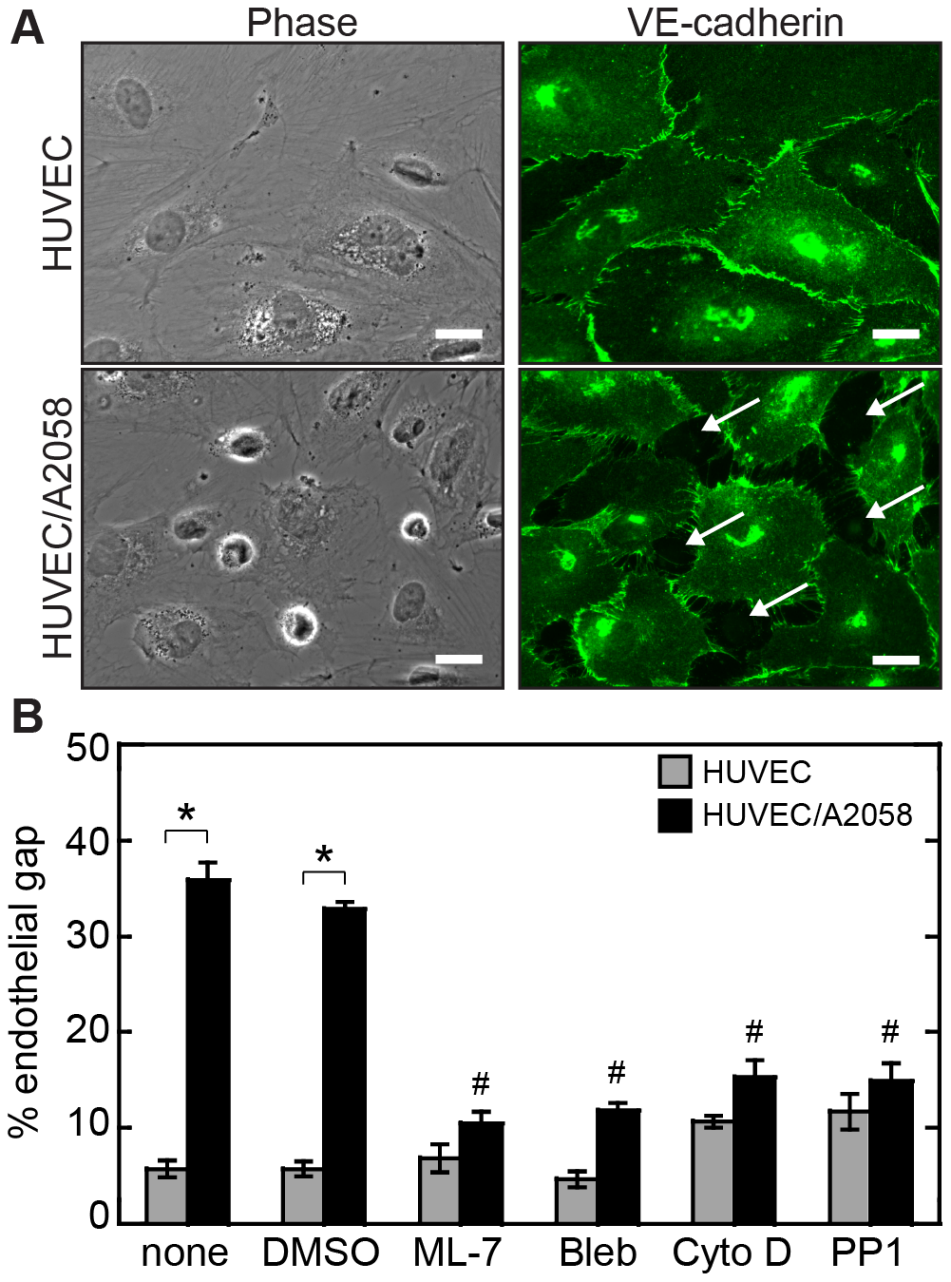
Co-culture of endothelial monolayers with A2058 melanoma cells induces endothelial cell-cell junction disruption local to melanoma cell adhesion sites. (A) Phase contrast images of HUVEC monolayers with corresponding immunofluorescence staining of VE-cadherin junctions. VE-cadherin staining indicates existence of cell-cell junction integrity in the endothelial monolayer. With A2058 co-culture, VE-cadherin staining shows dissolution of cell-cell junctions. White arrows indicate the location of A2058 melanoma cells. (B) Quantification of the percentage gap formation between endothelial cells with A2058 melanoma cell co-culture with and without inhibitors of cellular contractility, actin remodeling, and Src kinase signaling. *p<0.001 in comparison to HUVEC only controls. #p<0.001 in comparison to HUVEC/A2058 co-culture sample incubated with DMSO. Scale bars, 25 µm.

Melanoma cell signals including soluble cytokines and cell surface adhesive molecules can impact the barrier properties of the vascular endothelium. To examine how melanoma cues affect the integrity of endothelial cell-cell junctions, A2058 melanoma cells were co-cultured with HUVEC monolayers for 45 minutes. Cell-cell junctions were monitored by examining VE-cadherin localization and it was observed that co-culture of melanoma cells with HUVECs resulted in dissolution of VE-cadherin junctions local to the tumor cells (Figure 1A). Quantification of the area percentage of gaps between endothelial cells revealed a significant increase in gap percentage with co-culture of HUVEC monolayers and melanoma cells in comparison to HUVEC monolayers alone (Figure 1B). These results are consistent with previous results that demonstrate a role for melanoma signals including soluble factors such as IL-8 and IL-1β and receptor-ligand interactions between proteins displayed on the surfaces of tumor and endothelial cells such as VLA-4/VCAM-1 as mediators of gap formation [26], [34].

To examine the roles of endothelial cellular contractility, junction disassembly, and actin remodeling in melanoma-mediated gap formation, we experimentally targeted proteins within endothelial cells linked to these outputs. Cellular contractility is controlled by the actomyosin cytoskeleton, which is regulated by signaling through the Rho GTPases and MLCK. Decreasing cellular contractility through treatment with the non-muscle myosin ATPase inhibitor blebbistatin significantly reduced A2058-induced gap formation in HUVEC monolayers (Figure 1B). Cytochalasin D, an inhibitor of actin polymerization and remodeling, also decreased gap formation. VE-cadherin junction disassembly is mediated in part by phosphorylation of the cytoplasmic tail of VE-cadherin by Src kinase which leads to a decrease in the strength of junctions [6]. Inhibition of Src signaling by treatment with PP1 also resulted in reduced gap formation (Figure 1B). Although all three inhibitors significantly reduced gap formation, there was no significant difference between the blebbistatin, PP1, and cytochalasin D co-culture cases. These results suggest that actin remodeling, cellular contractility, and junction disassembly are all critical partners in gap formation.

### Model simulations reveal that cytoskeletal contractility followed by VE-cadherin phosphorylation initiates junction disassembly

**Figure 2:**
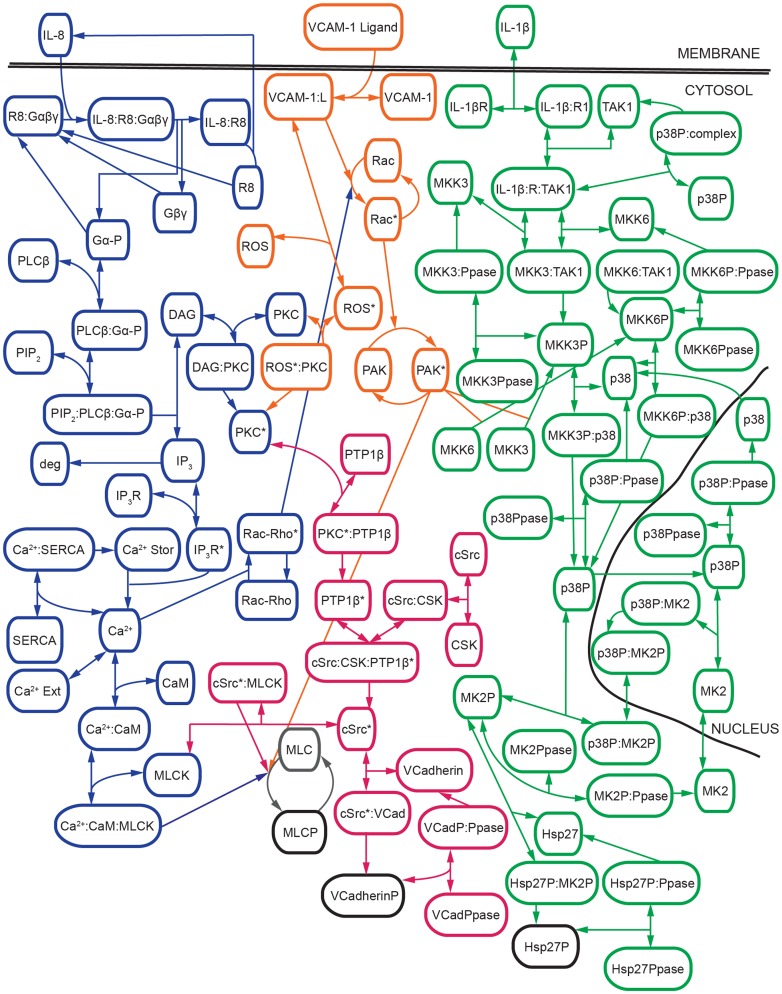
Schematic of network model connections. Pathways are distinguished by color with IL-8 in blue, VCAM-1 in orange, IL-1β in green, c-Src in magenta, and model outputs in black. Grey indicates locations where all pathways converge. Phosphorylated proteins are indicated with the letter P and protein activation is designated with an asterisk.

To examine the dynamic response of the endothelium to multiple melanoma cell signals simultaneously, we developed a model of the signaling networks involved in melanoma-induced gap formation. The model included melanoma cell signals (VCAM-1 ligand, IL-8, and IL-1β) as well as downstream p38 MAPK, Src, PKC, and MLC signaling pathways within the endothelium (Figure 2). The signaling cascades were altered to represent enzyme kinetics and a system of ordinary differential equations was developed using these kinetics. The signaling map in Figure 2 shows all of the signaling cascades within the model connecting the individual inputs to their respective outputs based on previous literature and experimental analysis [26], [34], [35].

To investigate the relative timing of activation of signaling cascades leading to actin remodeling, cellular contraction, and junction disassembly we monitored the levels of phosphorylated heat shock protein (HSP)-27, MLC, and VE-cadherin, respectively, within the simulation. Turning on all three inputs, VCAM-1, IL-8, and IL-1β, represented the melanoma cell stimulus. Previous studies have linked disruption of VE-cadherin junctions to phosphorylation of VE-cadherin and reduced junction strength [5], [6]; therefore, we used phosphorylated VE-cadherin (pVE-cadherin) as a marker of junction disassembly. HSP-27, a molecule that regulates actin/myosin cross-bridges, can be activated by p38 MAPK. Studies suggest that phosphorylation of HSP-27 enables the interaction between actin and myosin through its enhanced interactions with tropomyosin [36]. Activation of the p38MAPK/HSP-27 pathway is involved in the cellular response to stress from proinflammatory cytokines such as platelet-derived growth factor (PDGF), IL-1β, and transforming growth factor (TGF)-β [37]. Thus, we used phosphorylated HSP-27 (pHSP-27) as a marker of actin remodeling. Furthermore, phosphorylation of MLC results in increased cellular contractility and was thus used as a marker of actinomyosin contraction within the simulations.

**Figure 3:**
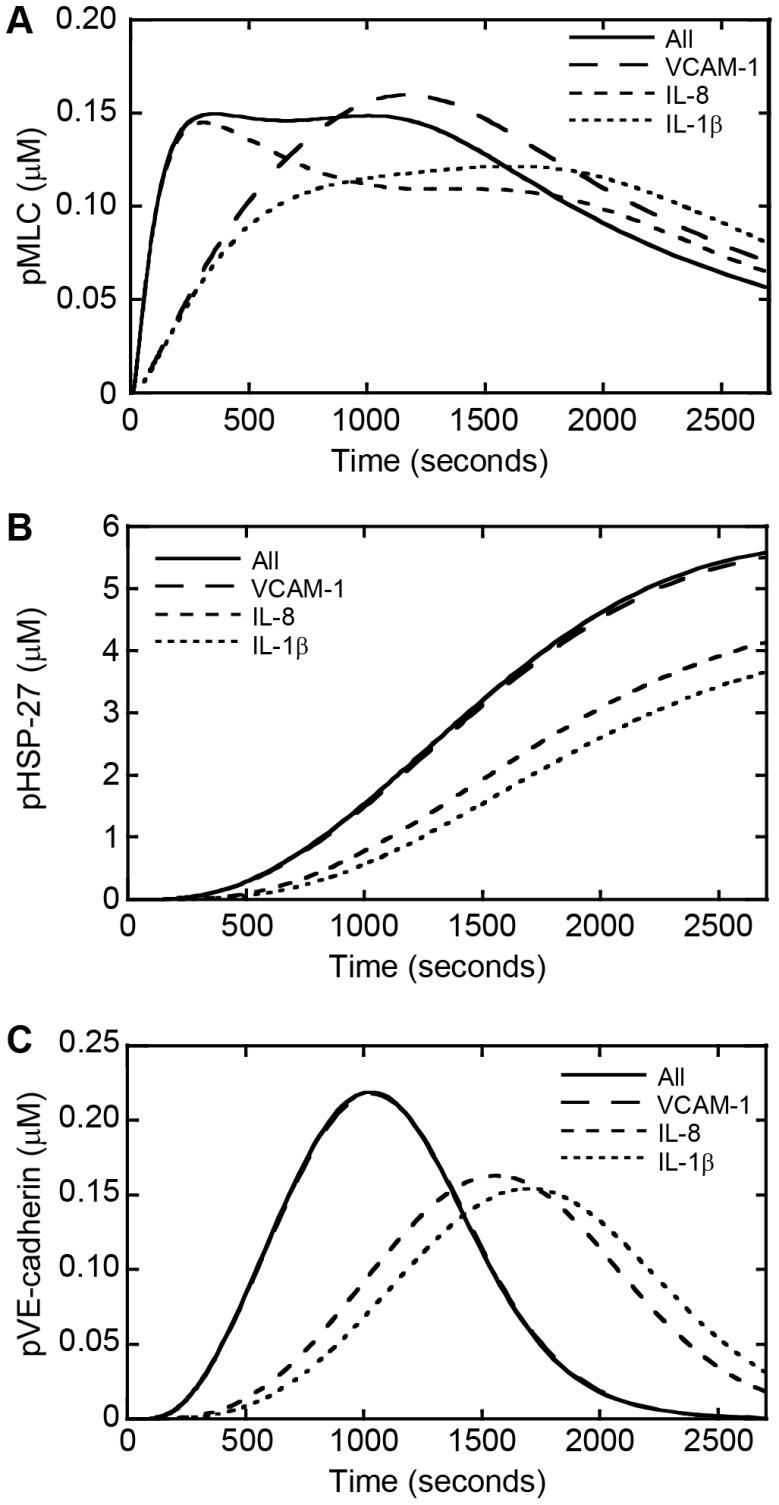
Computational simulations show that melanoma signals differentially affect endothelial contractility, actin remodeling, and junction disassembly. Phosphorylation dynamics as a function of time for (A) MLC, (B) HSP-27, and (C) VE-cadherin following activation of VCAM-1, IL-8, and IL-1β individually or simultaneous activation of all inputs together.

The time courses of each output were viewed over 45 minutes (2700 seconds) since melanoma-induced endothelial gap formation is observed to occur over 45 minutes experimentally (Figure 1). With all inputs turned on, the phosphorylation of MLC occurred very quickly with a peak concentration occurring at 300 seconds which continued to approximately 1300 seconds before slowly decreasing (Figure 3A). Phosphorylated HSP-27 took longer to activate as its concentration slowly increased over time (Figure 3B). The peak concentration of pVE-cadherin occurred towards the latter part of MLC activation around 1000 seconds (Figure 3C). These results suggest that when all input signals are activated that actinomyosin contraction and junction disassembly may work together at the beginning of gap formation, with actinomyosin contraction initiating the event, while actin remodeling occurs progressively through the time course and peaks toward the end of gap formation.

### Model simulations reveal a multifaceted interplay of VCAM-1, IL-8, and IL-1β signaling during endothelial gap formation

To determine whether VCAM-1, IL-8, and IL-1β have an additive, synergistic, or antagonistic effect on endothelial gap formation we also performed simulations with VCAM-1, IL-8, and IL-1β signaling pathways individually activated and compared these results to simultaneous activation of all three inputs. Each simulation was performed for 2700 seconds and the concentrations of pMLC, pHSP-27, and pVE-cadherin were monitored as a function of time. At early time points, pMLC dynamics associated with all inputs on was recapitulated by IL-8 alone suggesting that the IL-8 signaling cascade is likely responsible for the initial increase in pMLC levels within endothelial cells as well as for the induction of increased contractility. At intermediate and late time points, the case with all three inputs turned on did not produce the highest concentration of pMLC therefore suggesting antagonistic effects between the different individual signaling inputs (Figure 3A). For pHSP-27, the case with all three inputs activated produced the highest concentrations at all time points, but it was only marginally more than what is achieved by the VCAM-1 signaling cascade alone for each time point (Figure 3B). This suggests that the inputs are neither additive nor synergistic in their effects on actin remodeling. Furthermore, activation of all three inputs and VCAM-1 only produced similar time courses for pVE-cadherin concentration while IL-8 and IL-1β produced time courses with lower peak concentrations that occurred at a later time (Figure 3C). These data suggest that signaling through the VCAM-1 pathway dominates phosphorylation dynamics of HSP-27 and VE-cadherin while the dynamics of the phosphorylation of MLC is influenced by IL-8 at early time points and all three input signaling pathways at later time points.

### Inhibiting regulators of actin remodeling, cellular contractility, and junction disassembly affects endothelial gap formation

**Figure 4:**
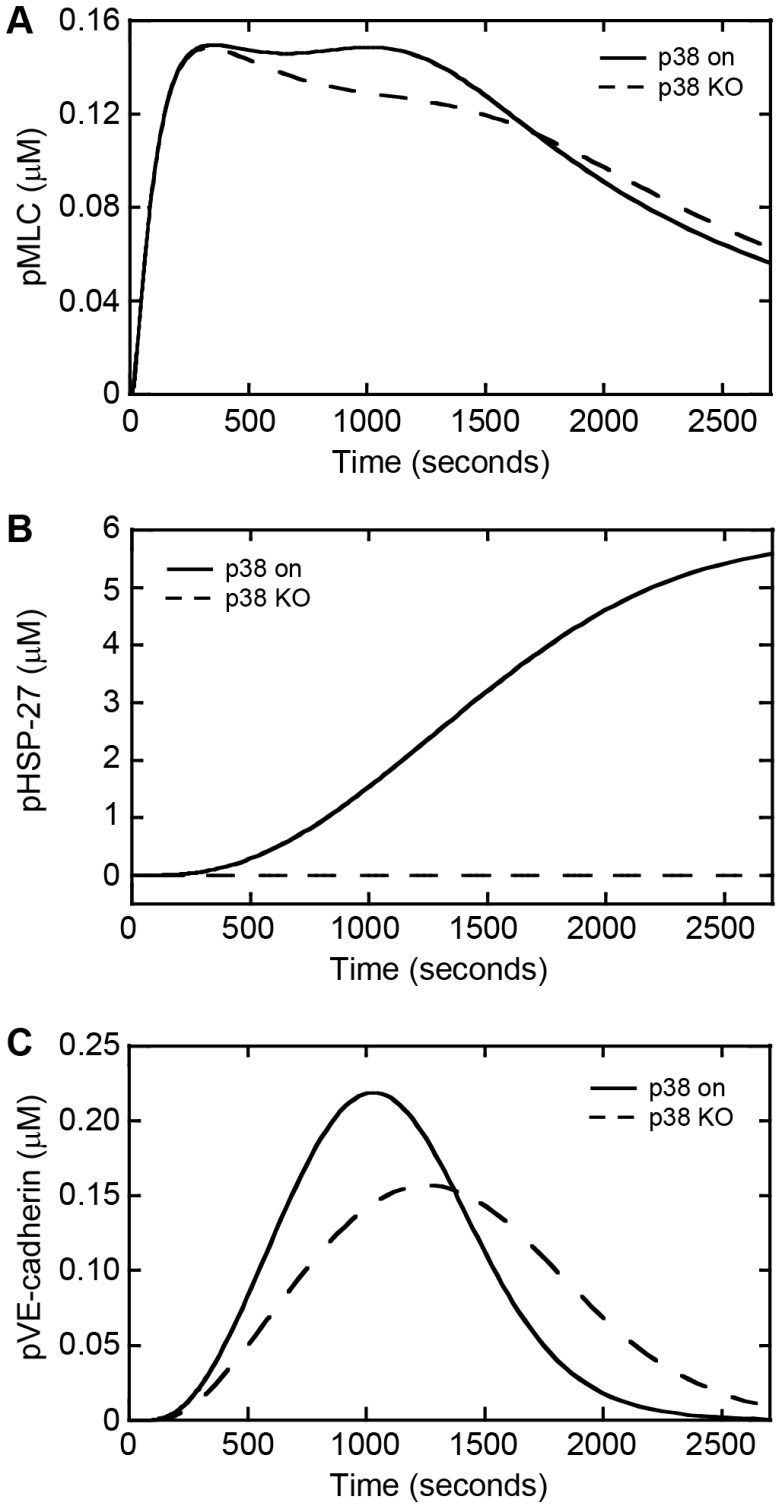
Simulations reveal that knockout of p38 MAPK influences MLC and VE-cadherin phosphorylation dynamics and completely inhibits HSP-27 phosphorylation. Phosphorylation dynamics as a function of time for p38 on and knockout of p38 for (A) MLC, (B) HSP-27, and (C) VE-cadherin.

We next investigated the effects of p38 MAPK, MLCK, and PKC signaling on gap formation since these molecules are influential regulators of actin remodeling, actinomyosin contraction, and junction disassembly, respectively [4], [38], [39]. Our previous studies have demonstrated an important role for p38 MAPK signal transduction in melanoma-induced endothelial cell-cell junction disruption and in experimental metastasis [34]. To further elucidate how p38 signal transduction affects actin remodeling, cellular contractility, and junction disassembly, we abrogated p38 signaling within the simulations while leaving MLC and PKC signaling intact (Figure 4A–C). The dynamics of MLC phosphorylation were affected minimally by p38 knock-out at early time points. In contrast, at later time points the concentration of pMLC exhibited a slow decrease over time as opposed to a sustained peak concentration followed by a decrease when p38 signaling was intact (Figure 4A). Inhibiting p38 signaling resulted in complete inhibition of the phosphorylation of HSP-27 (Figure 4B). This result is consistent with previous studies that demonstrate that p38 activates HSP-27 after stress and growth factor incubation and that inhibition of p38 blocks HSP-27 phosphorylation [40]. For pVE-cadherin, inhibition of p38 decreased the peak concentration by 32% while also delaying the time at which the peak level of pVE-cadherin is reached by approximately 200 seconds (Figure 4C). These data suggest that p38 MAPK signaling influences the dynamics of cellular contractility, actin remodeling, and junction disassembly within endothelial cells.

**Figure 5:**
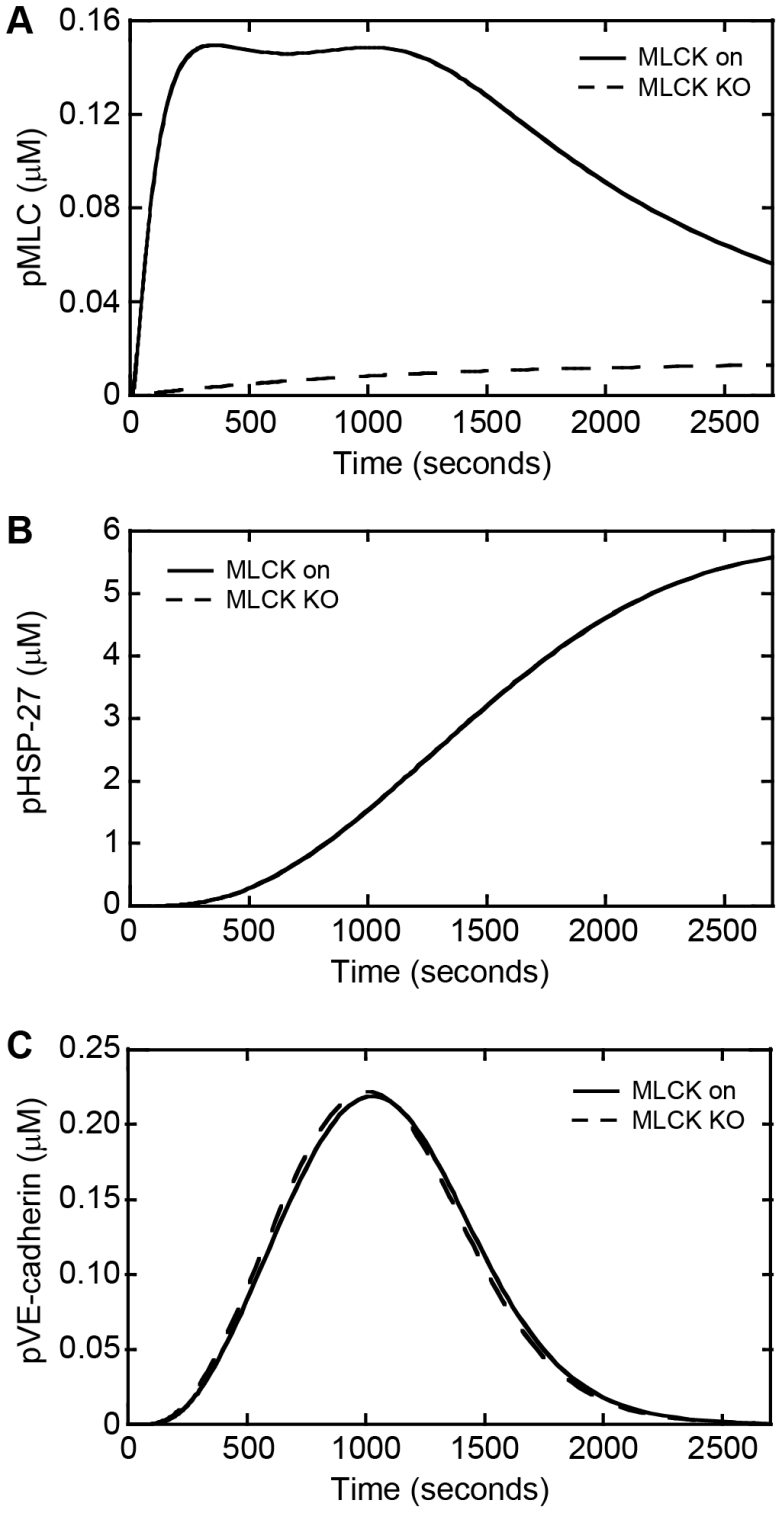
Simulations show that knockout of MLCK reduces MLC phosphorylation, whereas HSP-27 and VE-cadherin phosphorylation is minimally affected. Phosphorylation dynamics as a function of time for MLCK on and knockout of MLCK for (A) MLC, (B) HSP-27, and (C) VE-cadherin.

Given the important role of MLCK in controlling cellular contractility, we next examined the role of MLCK in regulating the dynamics of protein phosphorylation within endothelial cells in response to melanoma cues. Knockout of MLCK within the simulations resulted in a substantial decrease in the concentration of phosphorylated MLC across all time points although MLC phosphorylation was not completely abrogated (Figure 5A). Furthermore, the pHSP-27 and pVE-cadherin time courses remained nearly unchanged with MLCK inhibition in comparison to activated MLCK (Figure 5B,C). To experimentally test if melanoma cells induce signaling through the MLCK pathway within endothelial cells to contribute to gap formation, we treated HUVEC monolayers with the MLCK inhibitor ML-7 for 30 minutes prior to co-culture with A2058 melanoma cells. Treatment with ML-7 resulted in a significant decrease in A2058 melanoma-induced gap formation between endothelial cells thus confirming the importance of MLCK signaling in melanoma-mediated endothelial junction disruption (Figure 1B). Together these data suggest that MLCK influences gap formation through regulating cellular contractility, but has little effect on the dynamics of actin remodeling and junction disassembly represented by the levels of pHSP-27 and pVE-cadherin.

**Figure 6:**
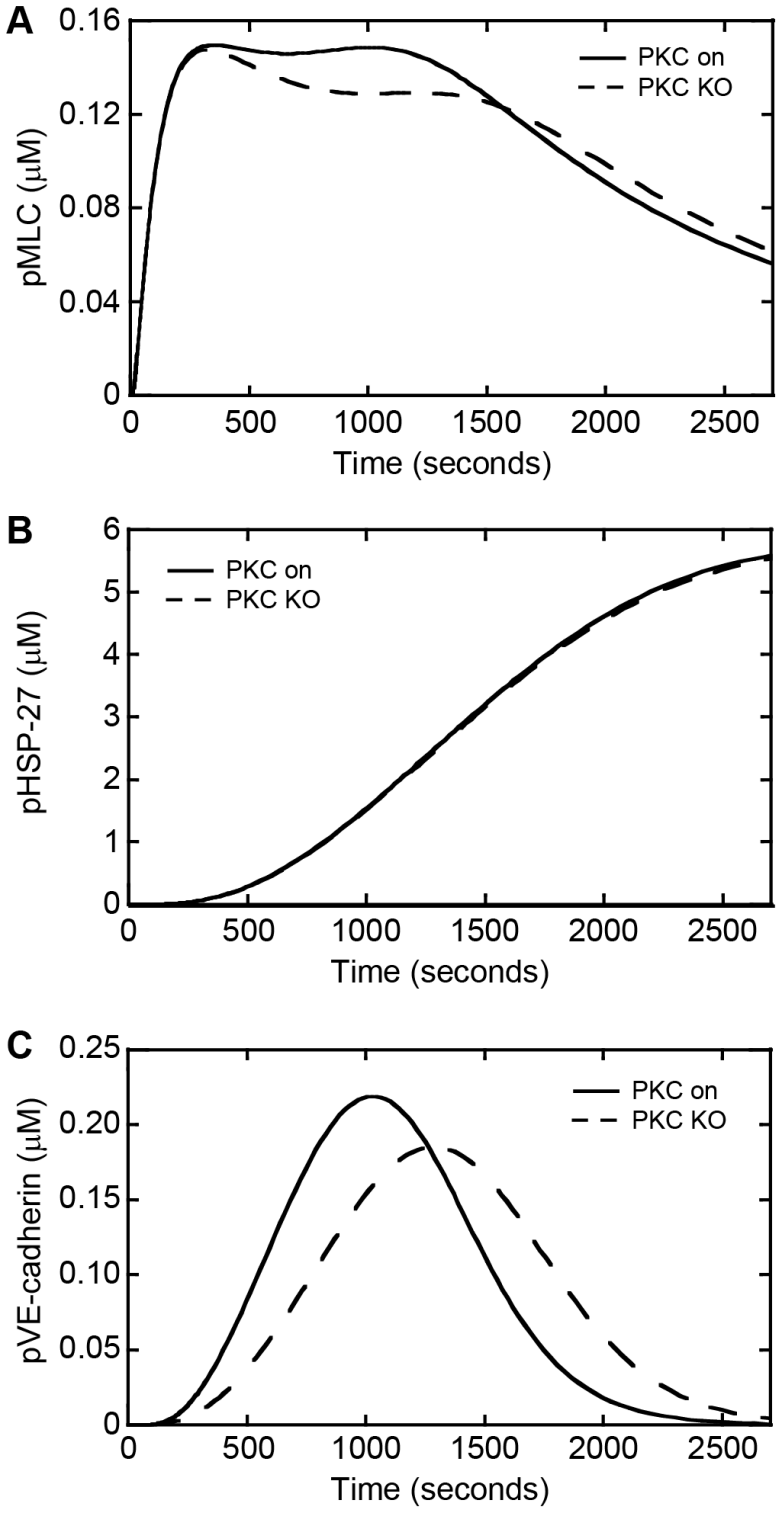
Knockout of PKC affects the simulated dynamics of MLC and VE-cadherin phosphorylation. Phosphorylation dynamics as a function of time for MLCK on and knockout of MLCK for (A) MLC, (B) HSP-27, and (C) VE-cadherin.

Signaling through PKC has been linked to endothelial barrier dysfunction and transendothelial migration [41], [42], [43], thus we next sought to determine how PKC affects cellular contractility, actin remodeling, and junction disassembly during melanoma-induced gap formation. Knockout of PKC within the simulations resulted in minimal effects on the level of pMLC at early time points with the same peak concentration being achieved (Figure 6A). A slow decrease in concentration was observed instead of a sustained peak concentration as observed in the control (Figure 6A). Inhibition of PKC produced an identical time course to when PKC signaling was intact for pHSP-27 (Figure 6B). There was a 23% decrease in the peak concentration of pVE-cadherin and a delayed peak concentration by 250 seconds compared to the control (Figure 6C). These data suggest that PKC modulates cellular contractility and junction disassembly but has no direct effect on actin remodeling.

## Discussion

In this study, we examined the roles of the melanoma cell inputs, IL-8, IL-1β, and VCAM-1 activation, and the endothelial cell outputs, actinomyosin contraction, junction disassembly, and actin remodeling, in melanoma-induced endothelial cell-cell junction breakdown. Actinomyosin contraction, junction disassembly, and actin remodeling have all been correlated with increased gap formation, but the importance that each output possesses in gap formation and whether there is synergy between the three to optimize gap formation is not clear. Experimentally, we demonstrated the importance of key signaling proteins in melanoma-induced gap formation. We also created an intracellular signaling network to connect the three inputs and their respective signaling cascades to factors that are responsible for regulating endothelial junction breakdown. Within the simulations, we quantitatively monitored the roles of the inputs and outputs involved in gap formation. Moreover, through the simulations we investigated the signaling dynamics that coordinate endothelial junction breakdown. Overall, our data suggest that cellular contractility, junction disassembly, and actin remodeling are all significant contributors to melanoma-induced endothelial gap formation.

Since it is difficult to monitor the dynamics of multiple signaling cascades simultaneously in direct co-culture experiments, we developed a model of the interactions between melanoma cells and endothelial cells to enable examination of signaling dynamics. Using this model, we investigated the phosphorylation dynamics of MLC, HSP-27, and VE-cadherin as read-outs of cellular contractility, actin remodeling, and junction disruption, respectively. We found that over the 45 minute (2700 second) window for gap formation, that the concentration of pMLC is maximized at early time points (from 300 to 1300 seconds) followed by pVE-cadherin at approximately 1000 seconds with the concentration of pHSP-27 peaking at 2700 seconds. Interestingly, pMLC reaches its peak concentration around the same time that pHSP-27 and pVE-cadherin concentrations start their initial increase. This suggests that crosstalk between the signaling pathways may be important to gap formation with actinomyosin contraction or pMLC possibly impacting or promoting phosphorylation of HSP-27 and VE-cadherin, actin remodeling, and junction disassembly. Indeed, recent studies show that during transendothelial migration monocytes activate the HRas/Raf/MEK/ERK signaling cascade which induces MLC phosphorylation followed by Src-mediated phosphorylation of VE-cadherin [44]. In this monocyte endothelial cell co-culture system, recruitment of Src to the VE-cadherin complex followed phosphorylation of MLC and inhibition of MLC attenuated VE-cadherin phosphorylation. Furthermore, α_2_β_1_ integrin displayed on the surface of breast cancer cells mediates phosphorylation of VE-cadherin in endothelial cells via Ras/MLC signaling [45]. These studies suggest a link between MLC phosphorylation and VE-cadherin phosphorylation via Src, however, it is currently not known whether an interplay between these factors exists in melanoma-induced disassembly of endothelial adherens junctions and if so what signaling molecules mediate this crosstalk. Further studies are necessary to clarify the crosstalk between these pathways.

Investigation of the role of the individual activation of VCAM-1, IL-8, and IL-1β signaling cascades revealed that the effects of these cues are neither additive nor synergistic on the phosphorylation dynamics of downstream signaling molecules, as activation of all inputs never resulted in additive effects or large increases in the levels of pMLC, pHSP-27, or pVE-cadherin over levels from individual activation during the time course of gap formation. The phosphorylation dynamics of HSP-27 and VE-cadherin resulting from VCAM-1 signaling alone most closely matched that produced by activation of all three pathways simultaneously suggesting that intracellular signaling cascades initiated by VCAM-1 likely have the most influence on actin remodeling and junction disassembly. Previous studies report that crosslinking VCAM-1 with antibodies is sufficient to induce stress fiber formation and gaps in HUVEC monolayers and blocking VCAM-1 activation on endothelial cells by monocytes through the use of specific antibodies attenuates phosphorylation of VE-cadherin [44]. Thus, melanoma-induced activation of VCAM-1 signaling may have similar effects. The phosphorylation dynamics of MLC with all three inputs activated are most closely matched at early times by IL-8 signaling alone with concentrations of pMLC at later times resulting from a complex interplay of the three inputs. This suggests that when the signaling cascades downstream of the three inputs are all active at the same time, the crosstalk between the pathways may interfere with rather than enhance activation of cellular contractility at later time points. Within these simulations, all three inputs were activated simultaneously, however, during melanoma-induced gap formation it is conceivable that the three inputs are not all activated within the endothelial cells at the exact same time and may in fact function with some type of delayed activation. Further studies exploring the idea of delayed activation between the three inputs will be informative.

Previous studies have shown that PKC, p38 MAPK, and MLCK are influential regulators of endothelial cell junction disassembly, actin remodeling, and actinomyosin contraction, respectively [4], [38], [39]. We wished to examine the effects of these intracellular signaling molecules on the phosphorylation dynamics of proteins within endothelial cells following exposure to melanoma cell cues. We find that knock-out of p38 MAPK within simulations affected the phosphorylation dynamics of MLC, HSP-27, and VE-cadherin. In contrast, knockout of MLCK only affected the phosphorylation of MLC while knockout of PKC affected the dynamics of both MLC and VE-cadherin phosphorylation. Our previous results and experimental data shown here confirm that inhibition of p38 MAPK and MLCK significantly reduces melanoma-induced gap formation [34]. We find that p38 MAPK affects all three outputs, therefore, it may be a more influential regulator of endothelial cell-cell junction breakdown than the other signaling molecules examined. Interestingly, studies have shown that the PKC and Src pathways may interact suggesting that targeting a single pathway may be ineffective due to redundancies [7]. Therefore, targeting downstream effectors like MLC may prove to be more promising therapeutic targets for blocking extravasation and metastasis due to inclusion of both upstream pathways.

This study focused on melanoma-induced endothelial cell-cell junction disruption, however, the results described may not be unique to the interaction between melanoma and the endothelium. Indeed, a variety of cancer cell types including melanoma [46], breast [47], [48], ovarian [49], [50], prostate [51], pancreatic [52], and bladder [53] cancer cells secrete IL-8. Furthermore, multiple cancer cell types including melanoma [19], [20], sarcoma [54], renal [55], ovarian [56], and lymphoma [57] are known to express VLA-4 on their surfaces which can mediate VCAM-1 signaling. Future investigations characterizing the cytokine secretion profiles and adhesion molecule expression of various tumor cells will be informative and may enable modeling similar to the studies presented here to shed light on important factors mediating tumor cell interactions with the endothelium and metastasis.

In summary, we present integrated experimental and computational analyses that show actinomyosin contraction, junction disassembly, and actin remodeling are all important factors in mediating melanoma-induced endothelial junction breakdown and we demonstrate that MLCK and p38 MAPK have significant roles in gap formation. Simulations showed that the peak levels of phosphorylated proteins associated with actinomyosin contraction and junction disassembly occur towards the beginning of gap formation while the peak level of phosphorylated proteins linked to actin remodeling occurs at later stages of gap formation. In addition, activating VCAM-1, IL-8, and IL-1β signaling cascades simultaneously has neither an additive nor synergistic effect on the concentration of phosphorylated proteins associated with contractility, junction disassembly, and actin remodeling individually in comparison to activation of each signaling pathway alone. Thus, melanoma-mediated endothelial junction disruption arises from a complex interplay of multiple tumor cell-associated signals as well as multiple intracellular signaling cascades within endothelial cells. Analysis of the interplay between these signaling pathways will enable a clearer understanding of how these cues act in concert to influence endothelial cell-cell junction breakdown and may suggest novel approaches for blocking cancer metastasis.

## Supporting Information

Table S1 **Initial concentrations (greater than zero) for signaling network model.**
(DOCX)

Table S2 **Kinetic parameters used within the signaling network model.**
(DOCX)

## References

[1] Sandig M, Voura EB, Kalnins VI, Siu CH (1997) Role of cadherins in the transendothelial migration of melanoma cells in culture. Cell Motil Cytoskel 38: 351–364. 10.1002/(SICI)1097-0169(1997)38:4<351::AID-CM5>3.0.CO;2-6 9415377

[2] Nicolson GL (1989) Metastatic tumor cell interactions with endothelium, basement membrane and tissue. Curr Opin Cell Biol 1: 1009–1019. 2697287 10.1016/0955-0674(89)90073-2

[3] Stroka KM, Konstantopoulos K (2014) Physical biology in cancer. 4. Physical cues guide tumor cell adhesion and migration. Am J Physiol Cell Physiol 306: C98–C109. 24133064 10.1152/ajpcell.00289.2013 PMC3919991

[4] Komarova Y, Malik AB (2010) Regulation of endothelial permeability via paracellular and transcellular transport pathways. Annu Rev Physiol 72: 463–493. 20148685 10.1146/annurev-physiol-021909-135833

[5] Vandenbroucke E, Mehta D, Minshall R, Malik AB (2008) Regulation of endothelial junctional permeability. Ann Ny Acad Sci 1123: 134–145. 18375586 10.1196/annals.1420.016

[6] Dejana E, Orsenigo F, Lampugnani MG (2008) The role of adherens junctions and VE-cadherin in the control of vascular permeability. J Cell Sci 121: 2115–2122. 18565824 10.1242/jcs.017897

[7] Tinsley JH, Wu MH, Ma WY, Taulman AC, Yuan SY (1999) Activated neutrophils induce hyperpermeability and phosphorylation of adherens junction proteins in coronary venular endothelial cells. J Biol Chem 274: 24930–24934. 10455168 10.1074/jbc.274.35.24930

[8] Vestweber D, Winderlich M, Cagna G, Nottebaum AF (2009) Cell adhesion dynamics at endothelial junctions: VE-cadherin as a major player. Trends Cell Biol 19: 8–15. 19010680 10.1016/j.tcb.2008.10.001

[9] Wessel F, Winderlich M, Holm M, Frye M, Rivera-Galdos R, et al (2014) Leukocyte extravasation and vascular permeability are each controlled in vivo by different tyrosine residues of VE-cadherin. Nat Immunol 15: 223–230. 24487320 10.1038/ni.2824

[10] Adam AP, Sharenko AL, Pumiglia K, Vincent PA (2010) Src-induced tyrosine phosphorylation of VE-cadherin is not sufficient to decrease barrier function of endothelial monolayers. J Biol Chem 285: 7045–7055. 20048167 10.1074/jbc.M109.079277 PMC2844154

[11] Amerongen GPV, van Delft S, Vermeer MA, Collard JG, van Hinsbergh VWM (2000) Activation of RhoA by thrombin in endothelial hyperpermeability - Role of Rho kinase and protein tyrosine kinases. Circ Res 87: 335–340. 10948069 10.1161/01.res.87.4.335

[12] Sun HR, Breslin JW, Zhu J, Yuan SY, Wu MH (2006) Rho and ROCK signaling in VEGF-induced microvascular endothelial hyperpermeability. Microcirculation 13: 237–247. 16627366 10.1080/10739680600556944

[13] Mierke CT (2011) Cancer cells regulate biomechanical properties of human microvascular endothelial cells. J Biol Chem 286: 40025–40037. 21940631 10.1074/jbc.M111.256172 PMC3220522

[14] Goeckeler ZM, Wysolmerski RB (1995) Myosin light-chain kinase-regulated endothelial-cell contraction - the relationship between isometric tension, actin polymerization, and myosin phosphorylation. J Cell Biol 130: 613–627. 7622562 10.1083/jcb.130.3.613 PMC2120532

[15] Garcia JGN, Davis HW, Patterson CE (1995) Regulation of endothelial-cell gap formation and barrier dysfunction - role of myosin light-chain phosphorylation. J Cell Physiol 163: 510–522. 7775594 10.1002/jcp.1041630311

[16] Shen Q, Wu MH, Yuan SY (2009) Endothelial contractile cytoskeleton and microvascular permeability. Cell Health Cytoskelet 2009: 43–50. 20871798 10.2147/chc.s5118 PMC2943648

[17] Shen Q, Rigor RR, Pivetti CD, Wu MH, Yuan SY (2010) Myosin light chain kinase in microvascular endothelial barrier function. Cardiovasc Res 87: 272–280. 20479130 10.1093/cvr/cvq144 PMC2895546

[18] Stockton RA, Schaefer E, Schwartz MA (2004) p21-activated kinase regulates endothelial permeability through modulation of contractility. J Biol Chem 279: 46621–46630. 15333633 10.1074/jbc.M408877200

[19] Garofalo A, Chirivi RGS, Foglieni C, Pigott R, Mortarini R, et al (1995) Involvement of the very late antigen-4 integrin on melanoma in interleukin-1 augmented experimental metastases. Cancer Res 55: 414–419. 7529137

[20] Liang S, Dong C (2008) Integrin VLA-4 enhances sialyl-Lewis(x/a)-negative melanoma adhesion to and extravasation through the endothelium under low flow conditions. Am J Physiol-Cell Ph 295: C701–C707. 10.1152/ajpcell.00245.2008 PMC2544447 18632734

[21] Klemke M, Weschenfelder T, Konstandin MH, Samstag Y (2007) High affinity interaction of integrin alpha4beta1 (VLA-4) and vascular cell adhesion molecule 1 (VCAM-1) enhances migration of human melanoma cells across activated endothelial cell layers. J Cell Physiol 212: 368–374. 17352405 10.1002/jcp.21029

[22] Peng HH, Hodgson L, Henderson AJ, Dong C (2005) Involvement of phospholipase C signaling in melanoma cell-induced endothelial junction disassembly. Front Biosci 10: 1597–1606. 15769649 10.2741/1643 PMC2782934

[23] Wennerberg K, Der CJ (2004) Rho-family GTPases: it's not only Rac and Rho (and I like it). J Cell Sci 117: 1301–1312. 15020670 10.1242/jcs.01118

[24] Wittchen ES (2009) Endothelial signaling in paracellular and transcellular leukocyte transmigration. Front Biosci 14: 2522–2545. 10.2741/3395 PMC2654604 19273217

[25] Bokoch GM (2003) Biology of the p21-activated kinases. Annu Rev Biochem 72: 743–781. 12676796 10.1146/annurev.biochem.72.121801.161742

[26] Khanna P, Weidert E, Vital-Lopez F, Armaou A, Maranas CD, et al (2011) Model simulations reveal VCAM-1 augment PAK activation rates to amplify p38 MAPK and VE-cadherin phosphorylation. Cell Mol Bioeng 4: 656–669.

[27] Hoch RC, Schraufstatter IU, Cochrane CG (1996) In vivo, in vitro, and molecular aspects of interleukin-8 and the interleukin-8 receptors. J Lab Clin Med 128: 134–145. 8765209 10.1016/s0022-2143(96)90005-0

[28] Waugh DJJ, Wilson C (2008) The interleukin-8 pathway in cancer. Clin Cancer Res 14: 6735–6741. 18980965 10.1158/1078-0432.CCR-07-4843

[29] Mierke CT, Zitterbart DP, Kollmannsberger P, Raupach C, Schlotzer-Schrehardt U, et al (2008) Breakdown of the endothelial barrier function in tumor cell transmigration. Biophys J 94: 2832–2846. 18096634 10.1529/biophysj.107.113613 PMC2267111

[30] Yano S, Nokihara H, Yamamoto A, Goto H, Ogawa H, et al (2003) Multifunctional interleukin-1beta promotes metastasis of human lung cancer cells in SCID mice via enhanced expression of adhesion-, invasion- and angiogenesis-related molecules. Cancer Sci 94: 244–252. 12824917 10.1111/j.1349-7006.2003.tb01428.x PMC11160152

[31] Sims JE, Smith DE (2010) The IL-1 family: regulators of immunity. Nat Rev Immunol 10: 89–102. 20081871 10.1038/nri2691

[32] Schmidt H, Jirstrand M (2006) Systems Biology Toolbox for MATLAB: a computational platform for research in systems biology. Bioinformatics 22: 514–515. 16317076 10.1093/bioinformatics/bti799

[33] Hendriks BS, Hua F, Chabot JR (2008) Analysis of mechanistic pathway models in drug discovery: p38 pathway. Biotechnol Prog 24: 96–109. 17918858 10.1021/bp070084g

[34] Khanna P, Yunkunis T, Muddana HS, Peng HH, August A, et al (2010) p38 MAP kinase is necessary for melanoma-mediated regulation of VE-cadherin disassembly. Am J Physiol-Cell Ph 298: C1140–C1150. 10.1152/ajpcell.00242.2009 PMC2867383 20181932

[35] Peng HH, Dong C (2009) Systemic analysis of tumor cell-induced endothelial calcium signaling and junction disassembly. Cell Mol Bioeng 2: 375–385. 19915693 10.1007/s12195-009-0067-5 PMC2776759

[36] Bitar KN (2002) HSP27 phosphorylation and interaction with actin-myosin in smooth muscle contraction. Am J Physiol Gastrointest Liver Physiol 282: G894–903. 11960785 10.1152/ajpgi.00141.2001

[37] Hedges JC, Dechert MA, Yamboliev IA, Martin JL, Hickey E, et al (1999) A role for p38MAPK/HSP27 pathway in smooth muscle cell migration. J Biol Chem 274: 24211–24219. 10446196 10.1074/jbc.274.34.24211

[38] Chang E, Heo KS, Woo CH, Lee H, Le NT, et al (2011) MK2 SUMOylation regulates actin filament remodeling and subsequent migration in endothelial cells by inhibiting MK2 kinase and HSP27 phosphorylation. Blood 117: 2527–2537. 21131586 10.1182/blood-2010-08-302281 PMC3062414

[39] Sandoval R, Malik AB, Minshall RD, Kouklis P, Ellis CA, et al (2001) Ca2+ signalling and PKC alpha activate increased endothelial permeability by disassembly of VE-cadherin junctions. J Physiol-London 533: 433–445. 11389203 10.1111/j.1469-7793.2001.0433a.x PMC2278647

[40] Guay J, Lambert H, Gingras-Breton G, Lavoie JN, Huot J, et al (1997) Regulation of actin filament dynamics by p38 map kinase-mediated phosphorylation of heat shock protein 27. J Cell Sci 110 Pt 3: 357–368. 9057088 10.1242/jcs.110.3.357

[41] Mine S, Tabata T, Wada Y, Fujisaki T, Iida T, et al (2002) Oxidized low density lipoprotein-induced LFA-1-dependent adhesion and transendothelial migration of monocytes via the protein kinase C pathway. Atherosclerosis 160: 281–288. 11849649 10.1016/s0021-9150(01)00582-2

[42] Siflinger-Birnboim A, Johnson A (2003) Protein kinase C modulates pulmonary endothelial permeability: a paradigm for acute lung injury. Am J Physiol Lung Cell Mol Physiol 284: L435–451. 12573983 10.1152/ajplung.00106.2002

[43] Ferro T, Neumann P, Gertzberg N, Clements R, Johnson A (2000) Protein kinase C-alpha mediates endothelial barrier dysfunction induced by TNF-alpha. Am J Physiol Lung Cell Mol Physiol 278: L1107–1117. 10835315 10.1152/ajplung.2000.278.6.L1107

[44] Haidari M, Zhang W, Chen Z, Ganjehei L, Warier N, et al (2011) Myosin light chain phosphorylation facilitates monocyte transendothelial migration by dissociating endothelial adherens junctions. Cardiovasc Res 92: 456–465. 21908648 10.1093/cvr/cvr240

[45] Haidari M, Zhang W, Caivano A, Chen Z, Ganjehei L, et al (2012) Integrin alpha2beta1 mediates tyrosine phosphorylation of vascular endothelial cadherin induced by invasive breast cancer cells. J Biol Chem 287: 32981–32992. 22833667 10.1074/jbc.M112.395905 PMC3463327

[46] Bar-Eli M (1999) Role of interleukin-8 in tumor growth and metastasis of human melanoma. Pathobiology 67: 12–18. 9873223 10.1159/000028045

[47] Lin Y, Huang R, Chen L, Li S, Shi Q, et al (2004) Identification of interleukin-8 as estrogen receptor-regulated factor involved in breast cancer invasion and angiogenesis by protein arrays. Int J Cancer 109: 507–515. 14991571 10.1002/ijc.11724

[48] Freund A, Chauveau C, Brouillet JP, Lucas A, Lacroix M, et al (2003) IL-8 expression and its possible relationship with estrogen-receptor-negative status of breast cancer cells. Oncogene 22: 256–265. 12527894 10.1038/sj.onc.1206113 PMC2034407

[49] Browne A, Sriraksa R, Guney T, Rama N, Van Noorden S, et al (2013) Differential expression of IL-8 and IL-8 receptors in benign, borderline and malignant ovarian epithelial tumours. Cytokine 64: 413–421. 23727325 10.1016/j.cyto.2013.05.006

[50] Wang Y, Xu RC, Zhang XL, Niu XL, Qu Y, et al (2012) Interleukin-8 secretion by ovarian cancer cells increases anchorage-independent growth, proliferation, angiogenic potential, adhesion and invasion. Cytokine 59: 145–155. 22579115 10.1016/j.cyto.2012.04.013

[51] Kim SJ, Uehara H, Karashima T, McCarty M, Shih N, et al (2001) Expression of interleukin-8 correlates with angiogenesis, tumorigenicity, and metastasis of human prostate cancer cells implanted orthotopically in nude mice. Neoplasia 3: 33–42. 11326314 10.1038/sj.neo.7900124 PMC1505029

[52] Xie K (2001) Interleukin-8 and human cancer biology. Cytokine Growth Factor Rev 12: 375–391. 11544106 10.1016/s1359-6101(01)00016-8

[53] Inoue K, Slaton JW, Kim SJ, Perrotte P, Eve BY, et al (2000) Interleukin 8 expression regulates tumorigenicity and metastasis in human bladder cancer. Cancer Res 60: 2290–2299. 10786697

[54] Mattila P, Majuri ML, Renkonen R (1992) VLA-4 integrin on sarcoma cell lines recognizes endothelial VCAM-1. Differential regulation of the VLA-4 avidity on various sarcoma cell lines. Int J Cancer 52: 918–923. 1281143 10.1002/ijc.2910520615

[55] Tomita Y, Saito T, Saito K, Oite T, Shimizu F, et al (1995) Possible significance of VLA-4 (alpha 4 beta 1) for hematogenous metastasis of renal-cell cancer. Int J Cancer 60: 753–758. 7896440 10.1002/ijc.2910600604

[56] Scalici JM, Harrer C, Allen A, Jazaeri A, Atkins KA, et al (2014) Inhibition of alpha4beta1 integrin increases ovarian cancer response to carboplatin. Gynecol Oncol 132: 455–461. 24378876 10.1016/j.ygyno.2013.12.031 PMC3939448

[57] Stroeken PJ, van Rijthoven EA, van der Valk MA, Roos E (1998) Targeted disruption of the beta1 integrin gene in a lymphoma cell line greatly reduces metastatic capacity. Cancer Res 58: 1569–1577. 9537266

