# Supplementary material for: Actinomyosin Contraction, Phosphorylation of VE-Cadherin, and Actin Remodeling Enable Melanoma-Induced Endothelial Cell-Cell Junction Disassembly
Source: PLoS One. 2014 Sep 16;9(9):e108092. doi: 10.1371/journal.pone.0108092 (PMC4167543; doi:10.1371/journal.pone.0108092)
Supplement: Table S1 — Initial concentrations (greater than zero) for signaling network model. (DOCX) [file pone.0108092.s001.docx]

| **Node** | **Initial Concentration (µM)** |
| --- | --- |
| *IL-8 Pathway* | |
| IL-8 | 15 |
| R_G | 18 |
| PLC | 18 |
| PIP2 | 18 |
| PKC | 1 |
| IP3 | 10 |
| Ca | 0.18 |
| SERCA | 10 |
| Ca_store | 1 |
| Ca_ext | 0.1 |
| CaM | 0.4 |
| MLCK | 1 |
| MLC | 1 |
| *VCAM-1 Pathway* | |
| anti_VCAM | 30 |
| VCAM | 1.25 |
| Rac | 0.1 |
| Rac_Rho | 0.1 |
| PAK | 0.1 |
| ROS | 1 |
| *IL-1β Pathway* | |
| IL-1β | 0.5 |
| IL-1βR | 1.25 |
| MKK3 | 0.05 |
| MKK6 | 0.065 |
| MKK3_phosphatase | 0.00433 |
| MKK6_phosphatase | 0.533 |
| p38 | 6.5 |
| p38_phosphatase | 1.61 |
| p38_phosphatase_nucleus | 0.00333 |
| MK2_nucleus | 105.5 |
| MK2_phosphatase | 0.138 |
| Hsp27 | 32.65 |
| Hsp27_phosphatase | 0.424 |
| *c-Src Pathway* | |
| PTP1β | 1 |
| cSrc_CSK | 1 |
| VCadherin | 1 |
| VCadherin_phosphatase | 0.1 |
