# Supplementary material for: Actinomyosin Contraction, Phosphorylation of VE-Cadherin, and Actin Remodeling Enable Melanoma-Induced Endothelial Cell-Cell Junction Disassembly
Source: PLoS One. 2014 Sep 16;9(9):e108092. doi: 10.1371/journal.pone.0108092 (PMC4167543; doi:10.1371/journal.pone.0108092)
Supplement: Table S2 — Kinetic parameters used within the signaling network model. (DOCX) [file pone.0108092.s002.docx]

| Parameter | Description | Value | Unit |
| --- | --- | --- | --- |
| *IL-8 Pathway* | | | |
| k1 | G protein/PLC association | 2.52 | μM^-1^s^-1^ |
| k2 | G protein/PLC dissociation | 1 | s^-1^ |
| k3 | PLC_G/PIP_2_ association | 0.83 | µM^-1^s^-1^ |
| k4 | PLC_G/PIP_2_ dissociation | 0.1 | s^-1^ |
| k5 | PIP_2__PLC_G → DAG + IP_3_ | 0.58 | s^-1^ |
| k6 | DAG → deg | 0.15 | s^-1^ |
| k7 | DAG/PKC association | 0.008 | µM^-1^s^-1^ |
| k8 | DAG/PKC dissociation | 8.6348 | s^-1^ |
| k9 | IP_3_ → deg | 2.5 | s^-1^ |
| k10 | IP_3_/IP_3_R association | 0.0022 | µM^-3^s^-1^ |
| k11 | IP_3_/IP_3_R dissociation | 1 | s^-1^ |
| k12 | IP_3_R → Ca^2+^ | 2.5 | s^-1^ |
| k13 | Ca^2+^/CaM association | 0.465 | µM^-4^s^-1^ |
| k14 | Ca^2+^/CaM dissociation | 10 | s^-1^ |
| k15 | Ca^2+^_CaM/MLCK association | 28 | µM^-1^s^-1^ |
| k16 | Ca^2+^_CaM/MLCK dissociation | 0.0308 | s^-1^ |
| k17 | Ca^2+^/SERCA association | 0.003 | µM^-1^s^-1^ |
| k18 | Ca^2+^/SERCA dissociation | 288 | s^-1^ |
| k19 | Ca^2+^ → Ca^2+^ store | 72 | s^-1^ |
| k20 | Ca^2+^_ext → Ca^2+^ | 0.005 | s^-1^ |
| k21 | R8:Gαβγ/IL-8 association | 1 | µM^-1^s^-1^ |
| k22 | R8:Gαβγ/IL-8 dissociation | 1 | s^-1^ |
| k23 | R8/IL-8 dissociation | 1 | s^-1^ |
| k24 | R8/Gαβγ association | 1 | µM^-1^s^-1^ |
| k25 | PKC activation | 1 | s^-1^ |
| k26 | Rac-RhoGDI activation | 1 | µM^-1^s^-1^ |
| k27 | Rac activation via PKC* | 1 | µM^-1^s^-1^ |
| k28 | Rac activation via Rac-RhoGDI* | 1 | µM^-1^s^-1^ |
| k29 | Ca^2+^ → Ca^2+^ ext | 0.00277 | s^-1^ |
| k30 | Rac-RhoGDI deactivation | 1 | s^-1^ |
| k31 | MLC phosphorylation via Ca^2+^:CaM:MLCK | 0.000183 | µM^-1^s^-1^ |
| k32 | MLC dephosphorylation | 0.000877 | s^-1^ |
| *VCAM-1 Pathway* | | | |
| k33 | VCAM-1:L association | 0.000756 | µM^-1^s^-1^ |
| k34 | VCAM-1:L dissociation | 0.018225 | s^-1^ |
| k35 | VCAM catalytic rate | 0.091231 | s^-1^ |
| k36 | Michaelis constant for Rac activation | 0.249838 | - |
| k37 | Rac catalytic rate | 0.091231 | s^-1^ |
| k38 | Michaelis constant for PAK activation | 0.249838 | - |
| k39 | PAK catalytic rate for MKK6 | 0.091231 | s^-1^ |
| k40 | Michaelis constant for MKK6 phosphorylation | 0.249838 | - |
| k41 | PAK catalytic rate for MKK3 | 0.091231 | s^-1^ |
| k42 | Michaelis constant for MKK3 phosphorylation | 0.249838 | - |
| k43 | Rac* deactivation | 1 | s^-1^ |
| k44 | PAK* deactivation | 1 | s^-1^ |
| k45 | MLC phosphorylation for PAK* | 0.1 | s^-1^ |
| k46 | VCAM-1:L/ROS association | 0.1 | µM^-1^s^-1^ |
| k47 | VCAM-1:L/ROS dissociation | 0.1 | s^-1^ |
| k48 | VCAM-1:L_ROS catalytic rate | 0.1 | s^-1^ |
| k49 | ROS*/PKC association | 0.1 | µM^-1^s^-1^ |
| k50 | ROS*/PKC dissociation | 0.1 | s^-1^ |
| k51 | ROS*_PKC catalytic rate | 0.1 | s^-1^ |
| *IL-1β Pathway* | | | |
| k52 | IL-1β:R1 → TAK1 complex activation | 0.000756 | s^-1^ |
| k53 | IL-1β:R1/TAK1cx degradation | 0.048004 | s^-1^ |
| k54 | IL-1β:R1/TAK1cx inactivation | 0.018225 | s^-1^ |
| k55 | TAK1cx reactivation | 0.013939 | s^-1^ |
| k56 | IL-1β/IL-1βR association | 0.001 | µM^-1^s^-1^ |
| k57 | IL-1β/IL-1βR dissociation | 0.002436 | s^-1^ |
| k58 | TAK1cx/p38P association | 1.35 | µM^-1^s^-1^ |
| k59 | TAK1cx/p38P dissociation | 0.32468 | s^-1^ |
| k60 | TAK1cx:p38P catalytic rate | 28.211 | s^-1^ |
| k61 | TAK1cx/MKK6 association | 0.087207 | µM^-1^s^-1^ |
| k62 | TAK1cx/MKK6 dissociation | 0.000858 | s^-1^ |
| k63 | TAK1cx:MKK6 catalytic rate | 4.7865 | s^-1^ |
| k64 | MKK6P/Phosphatase_MKK6_ association | 1 | µM^-1^s^-1^ |
| k65 | MKK6P/Phosphatase_MKK6_ dissociation | 0.001014 | s^-1^ |
| k66 | Phosphatase_MKK6_ catalytic rate | 27.039 | s^-1^ |
| k67 | TAK1cx/MKK3 association | 0.022793 | µM^-1^s^-1^ |
| k68 | TAK1cx/MKK3 dissociation | 0.091231 | s^-1^ |
| k69 | TAK1cx:MKK3 catalytic rate | 2.6008 | s^-1^ |
| k70 | MKK3P/Phosphatase_MKK3_ association | 1 | µM^-1^s^-1^ |
| k71 | MKK3P/Phosphatase_MKK3_ dissociation | 0.24677 | s^-1^ |
| k72 | Phosphatase_MKK3_ catalytic rate | 0.69753 | s^-1^ |
| k73 | MKK6P/p38 association | 1 | µM^-1^s^-1^ |
| k74 | MKK6P/p38 dissociation | 0.001938 | s^-1^ |
| k75 | MKK6P catalytic rate | 0.51072 | s^-1^ |
| k76 | MKK3P/p38 association | 1 | µM^-1^s^-1^ |
| k77 | MKK3P/p38 dissociation | 0.062731 | s^-1^ |
| k78 | MKK3P catalytic rate | 0.26937 | s^-1^ |
| k79 | p38P/Phosphatase_p38_ association (cytosol) | 1 | µM^-1^s^-1^ |
| k80 | p38P/Phosphatase_p38_ dissociation (cytosol) | 0.001919 | s^-1^ |
| k81 | Phosphatase_p38_ catalytic rate (cytosol) | 0.19203 | s^-1^ |
| k82 | p38P nuclear import rate | 0.40091 | s^-1^ |
| k83 | p38P/Phosphatase_p38_ association (nucleus) | 0.69 | µM^-1^s^-1^ |
| k84 | p38P/Phosphatase_p38_ dissociation (nucleus) | 0.011258 | s^-1^ |
| k85 | Phosphatase_p38_ catalytic rate (nucleus) | 0.056032 | s^-1^ |
| k86 | p38P nuclear export rate | 0.002797 | s^-1^ |
| k87 | p38 nuclear import rate | 0.000761 | s^-1^ |
| k88 | p38 nuclear export rate | 0.000313 | s^-1^ |
| k89 | p38P/MK2 association | 1 | µM^-1^s^-1^ |
| k90 | p38P/MK2 dissociation | 2.7272 | s^-1^ |
| k91 | p38P catalytic rate for MK2 | 16.198 | s^-1^ |
| k92 | p38P:MK2P nuclear import rate | 0.004514 | s^-1^ |
| k93 | p38P:MK2P nuclear export rate | 0.001832 | s^-1^ |
| k94 | p38P:MK2P dissociation | 1 | s^-1^ |
| k95 | MK2P/Phosphatase_MK2_ association | 1 | µM^-1^s^-1^ |
| k96 | MK2P/Phosphatase_MK2_ dissociation | 0.079075 | s^-1^ |
| k97 | Phosphatase_MK2_ catalytic rate | 10 | s^-1^ |
| k98 | MK2 nuclear import rate | 0.002311 | s^-1^ |
| k99 | MK2 nuclear export rate | 0.042338 | s^-1^ |
| k100 | MK2P/Hsp27 association | 1 | µM^-1^s^-1^ |
| k101 | MK2P/Hsp27 dissociation | 0.99823 | s^-1^ |
| k102 | MK2P catalytic rate | 1.0025 | s^-1^ |
| k103 | Hsp27P/Phosphatase_Hsp27_ association | 0.016189 | µM^-1^s^-1^ |
| k104 | Hsp27P/Phosphatase_Hsp27_ dissociation | 0.7796 | s^-1^ |
| k105 | Phosphatase_Hsp27_ catalytic rate | 10 | s^-1^ |
| *c-Src Pathway* | | | |
| k106 | PKC*/PTP1B association | 0.1 | µM^-1^s^-1^ |
| k107 | PKC*/PTP1B dissociation | 0.1 | s^-1^ |
| k108 | PKC*_PTP1B catalytic rate | 0.1 | s^-1^ |
| k109 | PTP1B*/cSrc_CSK association | 0.1 | µM^-1^s^-1^ |
| k110 | PTP1B*/cSrc_CSK dissociation | 0.1 | s^-1^ |
| k111 | cSrc_CSK_PTP1B* catalytic rate | 0.1 | s^-1^ |
| k112 | cSrc/CSK association | 0.1 | µM^-1^s^-1^ |
| k113 | cSrc/CSK dissociation | 0.1 | s^-1^ |
| k114 | cSrc*/MLCK association | 0.1 | µM^-1^s^-1^ |
| k115 | cSrc*/MLCK dissociation | 0.1 | s^-1^ |
| k116 | MLC phosphorylation via cSrc*_MLCK | 0.1 | s^-1^ |
| k117 | cSrc*/Vcadherin association | 0.1 | µM^-1^s^-1^ |
| k118 | cSrc*/Vcadherin dissociation | 0.1 | s^-1^ |
| k119 | cSrc*_Vcadherin catalytic rate | 0.1 | s^-1^ |
| k120 | pVCadherin/Phosphatase_VCadherin_ association | 0.1 | µM^-1^s^-1^ |
| k121 | pVCadherin/Phosphatase_VCadherin_ dissociation | 0.1 | s^-1^ |
| k122 | Phosphatase_VCadherin_ catalytic rate | 0.1 | s^-1^ |
| k123 | p38P/PTP1B association | 0.1 | µM^-1^s^-1^ |
| k124 | p38P/PTP1B dissociation | 0.1 | s^-1^ |
| k125 | p38P:PTP1B catalytic rate | 0.1 | s^-1^ |
